# Mesh use does not affect the outcome of elective open hernia repair in cirrhotic patients

**DOI:** 10.3389/fsurg.2025.1654774

**Published:** 2025-10-01

**Authors:** Fuat Aksoy, Burak Buyukpolat, Ozkan Balcin, Volkan Polatkan, Ekrem Kaya

**Affiliations:** 1 Department of Surgery, Bursa Uludag University, Bursa, Türkiye; 2 Department of Bioistatistic, Bursa Uludag University, Bursa, Türkiye

**Keywords:** mesh, cirrhosis, hernia, hernia (inguinal), hernia umbilical

## Abstract

**Purpose:**

The aim of the study is to evaluate the outcomes of elective and emergency umbilical and inguinal hernia repairs in cirrhotic patients, focusing on the controversial use of mesh and its relationship with the Model for End-Stage Liver Disease (MELD) score.

**Methods:**

A retrospective analysis of patients with open surgical hernia repair on cirrhosis between January 2007 and December 2022 was performed. Patients were divided into 2 main groups: those with and without mesh. Demographic characteristics, perioperative factors, postoperative complications, recurrence, and first 90-day mortality rates were compared.

**Results:**

Sixty-two patients were included in the study. Mesh use was more common in inguinal hernia repairs, while mesh-free repairs were predominant in umbilical hernias. No significant difference was observed in postoperative complications between the groups, but comorbidities were more common in the mesh group. Ascites and emergency vs. elective status did not significantly affect the decision to use mesh. No significant difference was found between the two groups in terms of recurrence. A positive correlation was determined between higher MELD scores and 90-day mortality, regardless of mesh use. (*p* < 0.01).

**Conclusions:**

Mesh can be safely used in hernia repairs in cirrhotic patients with appropriate perioperative management. However, it should be kept in mind that mortality may increase as the MELD score increases.

## Introduction

Liver cirrhosis, as a chronic liver disease characterized by extensive fibrosis and impaired hepatic function and mostly extensive ascitic fluid accommulation in abdominal cavity, may cause umbilical and inguinal hernias stand out as significant clinical concerns. The prevalence of hernias in individuals with cirrhosis is notably higher than in the general population, primarily attributed to increased intra-abdominal pressure, weakening of the abdominal wall musculature, and alterations in connective tissue integrity (1). These hernias not only diminish the patient's quality of life but also present a complex surgical scenario due to the compromised physiological state associated with cirrhosis.

Surgical repair of umbilical and inguinal hernias in cirrhotic patients necessitates understanding of the disease's pathophysiology and its implications on perioperative management. Patients who have chronic liver disease often exhibit coagulopathy, ascites, portal hypertension, and impaired hepatic metabolism of drugs, factors that significantly elevate the risks associated with surgical interventions (2, 3). For this reason, there are discussions in some studies about whether surgical repair or a “wait-and-see” approach should be applied (4, 5). Because even elective hernia repair carries a risk of morbidity and mortality in cirrhotic patients, some authors do not recommend it. Studies on this subject show that MELD and Child-Pugh classification may also be prognostic factors (6). On the other hand, some studies have shown that elective symptomatic hernias can be operated on in suitable patients with some risk models, even if they have a high MELD score (7).

Another controversial issue is recurrence after hernia repair especially patients having high intraabdominal pressure liked cirrhotic patients. Mesh use might be a solution for this problem but **ascitic leakage from the incision may be inevitable and may compromise patient comfort** and disturbs the patients comfort. Some previous studies showed that the use of mesh, especially in large defects, reduces recurrence rates, but it increases various complications such as surgical site infection, seroma, and adhesion in the postoperative period (8–10). It may also cause of ascites fluid leakeage from the hernia wound and compromises patient comfort.

The aim of the present study is to investigate the relationship between abdominal wall hernia repairs as a controversial issues of with or without mesh use and MELD score, and the postoperative results in patients with liver cirrhosis secondary to various etiologies.

## Patients and method

This retrospective data analysis includes patients who were operated on for inguinal and umbilical hernia between January 2007 and December 2022 and are followed up due to liver cirrhosis. Approval was received for the study by the ethics committee of Bursa Uludag University, **and the requirement for informed consent for retrospective data analysis was waived. However, all patients had provided written informed consent prior to surgery.** For the diagnosis of liver cirrhosis, patient history, physical examination, biochemical analysis, and radiological methods (CT, MRI, and ultrasound) were utilized. By scanning hospital medical records retrospectively, demographic characteristics of the patients, preoperative Model for End-stage Liver Disease (MELD) and CHILD-Pugh scores, etiology of cirrhosis, presence of ascites, whether it is elective or urgent surgery, other concomitant diseases, antimicrobial prophylaxis (AMP), use of drains, postoperative complications and mortality rates are analyzed. Cases who underwent transplantation due to liver cirrhosis were excluded from the study. All patients included in the study were operated on with open surgery. Spinal anesthesia was preferred for patients undergoing inguinal hernia repair, whereas general anesthesia was used for umbilical hernia procedures. Patients who underwent repair using mesh were fixed with onlay and 2/0 polypropylene suture as a standard procedure for umblical hernia. The standard approach to inguinal hernia repair was the Lichtenstein hernia repair procedure. Polypropylene mesh was used as standard for all patients in whom mesh was used. All patients were evaluated under 2 main headings: mesh used/not used. **According to the European Hernia Society (EHS) guidelines, extraperitoneal placement of mesh is recommended over intraperitoneal positioning due to lower risk of complications such as adhesion or fistula formation. Our findings align with this recommendation, as all mesh placements in our series were performed in an extraperitoneal fashion (**9**). Hernia defects were evaluated intraoperatively and categorized according to the European Hernia Society (EHS) classification system (**11**).**

Drain usage was decided based on intraoperative findings, particularly the presence and volume of ascitic fluid, as well as the anticipated risk of postoperative seroma or hematoma formation. Surgeons also considered individual patient characteristics and the potential for ascitic leakage. Drains were typically placed in patients with moderate to severe ascites or in cases where fluid accumulation was anticipated to facilitate postoperative care. **Definitions of complications and outcomes were standardized for consistency. An “ascitic fistula” was defined as persistent or recurrent leakage of ascitic fluid from the surgical wound for more than 48 h postoperatively. A “prolonged hospital stay” was defined as hospitalization exceeding 7 days after the procedure. Hernia recurrence was assessed through routine clinical examinations at 1, 3, 6, and 12 months postoperatively, and confirmed by radiological imaging (ultrasound or CT) in cases where clinical suspicion was present.**

Ascites severity was classified according to the criteria outlined by the European Association for the Study of the Liver (EASL) Clinical Practice Guidelines (12). Mild ascites was defined as detectable only by ultrasound, moderate ascites as moderate symmetrical abdominal distension, and severe ascites as marked abdominal distension with tense abdomen. This classification was used consistently in the assessment of patients throughout the study. **Preoperative ascites management was performed in all elective cases using diuretics (spironolactone and furosemide) and/or large-volume paracentesis when indicated. Elective surgeries were postponed until satisfactory ascites control was achieved. In contrast, emergency surgeries were performed without prior optimization, which may have contributed to the higher complication rates observed in this group.**

**Elective hernia repairs were generally scheduled 2–4 weeks after the initial surgical consultation, allowing time for preoperative optimization. Optimization strategies included diuretic therapy (spironolactone and furosemide), large-volume paracentesis when required, correction of coagulopathy (vitamin K and plasma transfusion if indicated), and nutritional support, including high-protein supplementation and albumin infusion when hypoalbuminemia was present.**

**Hernia defects were evaluated intraoperatively and categorized according to the European Hernia Society (EHS) classification system. For inguinal hernias, defect sizes were classified based on the medial and lateral EHS subgroups, while umbilical hernias were measured and categorized as small (<2 cm), medium (2–4 cm), or large (>4 cm). All inguinal hernia repairs were performed using the Lichtenstein tension-free technique with onlay placement of polypropylene mesh (average size approximately 8** **×** **12 cm). Umbilical hernia repairs were performed using an onlay polypropylene mesh (average size approximately 6** **×** **8 cm), fixed with 2/0 polypropylene sutures. In cases where mesh was not used, primary fascial closure with interrupted non-absorbable sutures was performed. Ascitic fistula formation was defined as persistent or recurrent ascitic fluid leakage from the surgical wound lasting more than 48 h, and was carefully documented.**

**Postoperative follow-up was conducted clinically at 1, 3, 6, and 12 months, with additional imaging (ultrasound or CT) when recurrence was suspected.**

### Antimicrobial prophylaxis (AMP)

We routinely administer 2 g of intravenous cefazolin as empirical prophylaxis in such cases. However, the distribution of bacterial species in ascitic fluid and wound infections highlights the need for individualized antimicrobial strategies or empirical antibiotic treatment. Empirical antibiotic regimens commonly include third-generation cephalosporins or piperacillin-tazobactam, which are subsequently adjusted according to culture and susceptibility results. Infections involving methicillin-resistant Staphylococcus aureus (MRSA) are treated with vancomycin or linezolid, whereas infections caused by Pseudomonas aeruginosa necessitate the use of carbapenems or aminoglycosides.

### Statistical analysis

Statistical analysis were done with SPSS 22 (IBM Corp, Released 2012. IBM SPSS Statistics for Windows, Version 21.0. Armonk, NY9). Categorical variables were summarized with percentage. Variables were expressed as mean ± standard deviation or as median (minimum: maximum, range) values depending on whether the variable followed a normal distribution or not, using the Shapiro–Wilk normality test. The log-rank test was used to determine the difference between Kaplan–Meier curves for both overall survival (OS). Results were reported as hazard ratio with 95% confidence intervals (CI) and related *p*-values. Statistical significance was defined as *p* < 0.05. **Since this was a retrospective study, no *a priori* sample size calculation was performed. However, a *post-hoc* power analysis was conducted based on the observed difference in recurrence rates between the mesh (10.8%) and non-mesh (20.0%) groups. With our total sample size of 62 patients (mesh** **=** **42, non-mesh** **=** **20) and a two-sided *α*** **=** **0.05, the calculated statistical power was approximately 52.7%, indicating moderate ability to detect clinically relevant differences in recurrence rates.**

## Results

### Patient demographic and preoperative data

A total of 62 patients diagnosed with cirrhosis and operated on due to inguinal or umbilical hernia were included in the study. Demographic characteristics and etiologies of the patients according to mesh use are given in Table 1. While the most of the cases in which mesh was not used are umbilical hernia repairs, mesh was seen to be used more in inguinal hernia repairs. When the mesh and non-mesh groups were compared in terms of demographic characteristics, Statistically significant difference was not determined. It was observed that the majority of the patients included in the study had viral hepatitis etiology.

**Table 1:** Demographic characteristics of patients according to mesh use.

Variables	Mesh (−)	Mesh (+)	*p*-value
	(*n* = 20)	(*n* = 42)	
Hernia type			0.001^a^
Umbilical hernia	18	17	
Inguinal hernia	2	25	
Age	54.95 ± 12.7	60.52 ± 9.7	0.095^c^
Gender			0.029^d^
Female	12 (60%)	13 (31%)	
Male	8 (40%)	29 (69%)	
MELD score	14.5 (7–25)	12 (6–29)	0.156^b^
CHILD score			0.540^d^
A	7 (35%)	21 (50%)	
B	10 (50%)	16 (38.1%)	
C	3 (15%)	5 (11.9%)	
Etiology			0.343^e^
Hepatitis B/C	6 (30%)	21 (50%)	
Non-alcoholic steatohepatitis	2 (10%)	4 (9.5%)	
Cryptogenic	6 (30%)	7 (16.7%)	
Alcohol	1 (5%)	5 (11.9%)	
Other	5 (25%)	5 (11.9%)	

Data are expressed as *n* (%).

MELD: model for end stage liver disease.

a Chi-square test with Yates correction.

b Mann–Whitney *U Test.*

c Independent sample *t test*.

d Pearson Chi square test.

e Fisher-Freeman-Halton test.

### Operative data, postoperative complications and follow-up

This section provides a comparison of various postoperative factors between patients who underwent surgical procedures with mesh placement [Mesh ( + ), *n* = 42] and those without mesh placement [Mesh (–), *n* = 20]. The postoperative complications and perioperative factors analyzed including the presence of ascites, co-morbidities, type of surgery (elective vs. urgent), duration of operation, use of antibiotic prophylaxis, anesthesia type, and drain status are shown in Table 2.

**Table 2:** Clinical and operative findings.

Variables	Mesh (−)	Mesh (+)	*p*-value
	(*n* = 20)	(*n* = 42)	
Ascites			0.544^c^
None	7 (35%)	19 (45.2%)	
Slight	5 (25%)	6 (14.3%)	
Moderate	8 (40%)	17 (40.5%)	
Co-morbidity			0.034^d^
None	7 (35%)	16 (38.1%)	
Diabetes Mellitus (DM)	10 (50%)	13 (31%)	
Hypertension	0 (0%)	8 (19%)	
Asthma	2 (10%)	0 (0%)	
Others	1 (5%)	5 (11.9%)	
Operation type			0.177^a^
Elective	9 (45%)	28 (66.7%)	
Urgent	11 (55%)	14 (33.3%)	
Duration of operation (min)	60 (40–120)	60 (30–140)	0.918^b^
Antibiotic prophylaxis			0.588^d^
None	2 (10%)	2 (4.8%)	
Yes	18 (90%)	40 (95.2%)	
Anesthesia type			0.711^a^
General	15 (75%)	28 (66.7%)	
Regional	5 (25%)	14 (33.3%)	
Drain status			0.655^a^
None	11 (55%)	19 (45.2%)	
Yes	9 (45%)	23 (54.8%)	

Data are expressed as *n* (%).

a Chi-square test with Yates correction.

b Mann–Whitney *U Test*.

c Pearson Chi square test.

d Fisher-Freeman-Halton test.

#### EHS classification of hernia defects

The distribution of umbilical and inguinal hernias according to the EHS classification is summarized in Table 3.

**Table 3:** Distribution of umbilical and inguinal hernias according to the European hernia society (EHS) classification.

Hernia type	Classification number of patients	Percentage	(%)
Umbilical	W1	8	24
	W2	18	53
	W3	8	23
Inguinal	L2/L3	20	80
	M2/M3	5	20
	Femoral	0	0

#### Operation type

The type of surgery was classified as either elective or urgent. In the mesh group, 28 patients (66.7%) underwent elective surgery, whereas 14 patients (33.3%) had urgent operations. In the non-mesh group, 9 patients (45.0%) underwent elective surgery and 11 patients (55.0%) underwent urgent intervention. Although elective procedures were more common in the mesh group, the difference in operation type between the two groups was not statistically significant (*p* = 0.177, Chi-Square test with Yates correction). Postoperative complications were significantly more frequent in patients undergoing emergency surgery. Ascitic fistula occurred in 10 of 25 patients (40%) who had emergency operations, compared to 4 of 37 patients (10.8%) who underwent elective surgery (*p* = 0.021). SSI was observed in 9 patients (36%) in the emergency group vs. 5 patients (13.5%) in the elective group (*p* = 0.038). Similarly, prolonged hospital stay (defined as >7 days) was noted in 14 patients (56%) in the emergency group, compared to 10 patients (27%) in the elective group (*p* = 0.043). These findings underscore the adverse impact of emergency surgery on postoperative outcomes in cirrhotic patients undergoing hernia repair.

#### Ascites fistula formation in mesh and non-mesh groups

The occurrence of ascites fistula following hernia repair in cirrhotic patients was analyzed in both the mesh and non-mesh groups. In total, 20 out of 42 patients (47.6%) in the mesh group developed an ascitic fistula, compared to 6 out of 20 patients (30.0%) in the non-mesh group. Although the incidence was numerically higher in the mesh group, the difference did not reach statistical significance (*p* = 0.299). However, patients with moderate ascites developed ascitic fistulas more frequently than those with mild ascites (56% vs. 32.1%, *p* = 0.038). (Table 4). Drain usage was also higher in patients who developed ascitic fistulas (*p* = 0.038). Additionally, the average hospital stay was longer in patients with ascitic fistula formation (9.5 ± 3.2 days vs. 6.3 ± 2.7 days, *p* = 0.041).

**Table 4:** Postoperative complications and follow-up.

Variables	Mesh (−)	Mesh (+)	*p*-value
	(*n* = 20)	(*n* = 42)	
Ascites fistula			0.299^a^
No	14 (70%)	22 (52,4%)	
Yes	6 (30%)	20 (47.6%)	
Other complications			0.705^b^
None	20 (100%)	34 (81%)	
Superficial ıncisional SSI	0 (0%)	4 (9.5%)	
Deep ıncisional SSI	0 (0%)	1 (2.4%)	
Acute renal failure	0 (0%)	1 (2.4%)	
Intracranial hemorrage	0 (0%)	1 (2.4%)	

Data are expressed as n(%).

a Chi-Square test with Yates correction.

b Fisher-Freeman-Halton test.

#### Microbiological analysis of ascitic fluid and surgical site infections

Four patients (9.5%) in the mesh group developed a superficial surgical site infection (SSI), while none of the patients in the non-mesh group experienced this complication. Deep incisional SSI occurred in 1 patient (2.4%) in the mesh group, but none in the non-mesh group. Although the complication rates were slightly higher in the mesh group, the differences did not reach statistical significance (*p* = 0.705, Fisher-Freeman-Halton Test) (Table 4). In this study, microbiological cultures were systematically obtained from all patients undergoing hernia repair, including both ascitic fluid and surgical site infection (SSI) samples. The results demonstrated that Escherichia coli was the most frequently isolated pathogen in ascitic fluid cultures, detected in 18 cases (29.0%), followed by Klebsiella pneumoniae in 12 cases (19.4%) and Enterococcus faecium in 10 cases (16.1%). For surgical site infections, Staphylococcus aureus was the most commonly identified organism, with methicillin-sensitive S. aureus (MSSA) found in 10 cases (16.1%) and methicillin-resistant S. aureus (MRSA) in 3 cases (4.8%). Additionally, Pseudomonas aeruginosa was cultured in 8 cases (12.9%), while Enterococcus faecalis was identified in 5 cases (8.1%). Notably, Enterococcus faecium, which was more commonly isolated from ascitic fluid, was also detected in 6 cases (9.7%) of SSI.

#### Hernia recurrence and survival

Among patients without mesh usage, 20 (80%) did not experience recurrence, while 5 (20%) had recurrence. In patients with mesh (+), 33 (89.19%) did not experience recurrence, while 4 (10.81%) had recurrence. The relationship between mesh usage, ascites fistula and MELD score and recurrence was analyzed using multivariate and univariate logistic regression, which showed no statistically significant association. In terms of first 90-day survival, mortality was found in 3 (15%) patients in the non-mesh group and 3 (7.1%) patients in the mesh group (statistically not significant). On the other hand, a positive correlation was found between the MELD score and the first 90-day mortality rates in patients using mesh. It was found that as the MELD score increased, mortality rates also increased significantly. In total, it was concluded that the increase in the MELD score was positively correlated with mortality in the first 90 days (Table 5).

**Table 5:** First 90-day survival analysis.

MELD score	Mesh (−)		Mesh (+)		Total	
	(*n* = 20)		(*n* = 42)		(*n* = 62)	
	r_s_	*p*-value	r_s_	*p*-value	r_s_	*p*-value
First 90 day mortality	0.39	0.089	0.448	**0**.**003**	0.431	**<0**.**001**

r_s_, Spearman correlation coefficient. Values in bold indicate statistical significance (*p* < 0.05).

## Discussion

The frequency and management of umbilical and inguinal hernias in cirrhotic patients is notably more controversial than the normal population. There is no clear consensus on elective or emergency hernia repair, timing, and use of mesh, especially in patients with ascites, high MELD score, or decompensated cirrhosis. It is also a fact that hernia development is more common in cirrhotic patients due to complications such as malnutrition, thrombocytopenia, and ascites (13). On the other hand, postoperative wound healing following hernia repair in cirrhotic patients may be impaired due to underlying hepatic dysfunction and the presence of ascites, given the increased frequency of complications such as infection, dehiscence, and ascitic leakage (14). In our study, we aimed to evaluate inguinal and umbilical hernia repairs performed in cirrhotic patients in a single center, by separating them with the use of mesh, which is considered the most controversial repair method, and to discuss the results regarding the timing or necessity of elective/emergency repairs in patients, especially by providing correlation with the MELD score.

The demographic characteristics of the mesh and non-mesh groups in the present study were comparable, and no significant differences were found in terms of patient age, gender, or comorbidities. This strengthens the validity of our findings, as the outcomes are unlikely to be influenced by baseline demographic differences. The use of mesh was more frequent in inguinal hernia repairs, while non-mesh repairs were more common in umbilical hernias (15). This distinction may reflect surgical preferences or concerns regarding the anatomical differences between these two types of hernias in cirrhotic patients, it may be depending on surgeons' habits and surgical trend for last three decades.

Ascites, traditionally viewed as a complicating factor in hernia repairs in cirrhotic patients, did not seem to influence the decision to use mesh in our study. This is an important finding, as it challenges the notion that ascites increases the risk of complications associated with mesh use. Similarly, the status of surgery as emergency or elective did not have a significant impact on mesh utilization (16, 17). This suggests that mesh can be safely considered even in urgent cases, provided that proper perioperative management is in place.

The lack of significant differences in other factors suggests that the use of mesh does not substantially impact the overall perioperative or postoperative outcomes, except in the context of pre-existing co-morbidities, where a more detailed analysis might be required to understand the clinical relevance of this finding. On the other hand, the results of the study revealed that the use of drains, particularly in patients with moderate ascites, led to the formation of ascitic fistulas and prolonged hospital stays. Therefore, the routine use of drains should be reconsidered. The ascites dranaige catheter insertion to the abdominal cavity instead of drain replacement in hernia incision may be more beneficial.

The comparison between elective and emergency hernia repair in cirrhotic patients remains a critical aspect of surgical decision-making, as the timing of intervention can significantly impact morbidity and mortality. In our study, 33.3% of mesh repairs and 55% of non-mesh repairs were performed urgently and the results demonstrated that patients undergoing emergency surgery had significantly higher rates of postoperative complications, including ascites fistula (*p* = 0.021), surgical site infections (*p* = 0.038), and prolonged hospital stays (*p* = 0.043) compared to those who underwent elective repairs. These findings align with previous studies (7). which reported that emergency hernia repairs in cirrhotic patients were associated with a higher risk of decompensation, sepsis, and mortality, particularly in those with MELD scores ≥15 (6). Similarly, elective hernia repair in cirrhotic patients with well-controlled ascites has been shown to have a significantly lower postoperative complication rate compared to emergency interventions (3). One of the major concerns in emergency repair is the high rate of perioperative morbidity, which is often linked to poor nutritional status, uncontrolled ascites, and coagulopathy. Previous studies (1, 18). support the notion that preoperative ascites control using diuretics or paracentesis reduces surgical risks and improves outcomes in elective repairs. Our study further corroborates this, as patients who underwent elective repair after preoperative ascites management had a lower rate of ascitic fistula formation (*p* = 0.034) and shorter hospital stays. The presence of ascites at the time of surgery remains a significant risk factor for postoperative leakage and infection. While mesh use did not independently increase the risk of ascitic fistula formation, careful patient selection, preoperative ascites control, and postoperative drainage management are essential to minimize complications.

Despite the risks associated with cirrhosis, recent literature suggests that elective hernia repair can be safely performed in well-selected patients (19). It has been emphasized that patients with MELD scores <12 and controlled ascites have outcomes comparable to non-cirrhotic patients when operated electively (14). However, in emergency settings, the risk of bowel strangulation, ischemia, and sepsis dramatically increases, leading to worse short-term outcomes (20). Our findings support the recommendation that whenever feasible, elective repair should be prioritized in cirrhotic patients to reduce surgical risks. Preoperative ascites management, nutritional optimization, and a multidisciplinary approach involving hepatology, anesthesia, and surgery are crucial in achieving better postoperative outcomes. Future studies with larger patient cohorts and prospective designs are needed to establish refined risk stratification models for selecting optimal surgical timing in this high-risk patient population.

Postoperative complications were not significantly different between the mesh and non-mesh groups. This result supports the safety of using mesh in cirrhotic patients and aligns with other studies showing that mesh can be beneficial in reducing hernia recurrence without exacerbating surgical complications (21). Ascites fistula was more frequent in the mesh group, but not to a statistically significant degree. Other complications, including infections, renal failure, hemorrhage, and death, were also more common in the mesh group, but the small sample sizes likely contributed to the lack of statistical power to detect meaningful differences. Additionally, the fact that the majority of patients received antibiotic prophylaxis likely contributed to the low rate of infections observed, highlighting the importance of infection control in this patient population (19). It is important to note that although the mesh group appeared to have a higher complication rate, especially concerning superficial SSIs, the overall *p*-value suggests that these differences could be due to random variation rather than a direct result of mesh use. This underlines the importance of further research with larger cohorts to confirm these findings and guide clinical decisions regarding mesh use in surgery. The high rate of bacterial growth in ascitic fluid and SSI cultures reinforces the need for perioperative infection control, particularly in cirrhotic patients undergoing hernia repair. The findings suggest that preoperative prophylactic antibiotic administration, strict aseptic surgical techniques, and close postoperative monitoring are essential for reducing infectious complications in this high-risk population.

One of the key findings of our study is the positive correlation between the MELD score and 90-day mortality rates in the mesh group. Although mesh use alone was not associated with an increase in mortality, higher MELD scores were predictive of worse outcomes (20). This suggests that patients with more advanced liver disease are at higher risk of mortality, regardless of the surgical technique used. Surgeons should therefore carefully consider the MELD score when planning hernia repairs in cirrhotic patients, as those with higher scores may require more intensive perioperative management and follow-up (18). **Based on our findings, patients with MELD ≥15 showed a markedly higher risk of 90-day mortality compared to those with lower scores. While our cohort size does not allow us to define a strict cutoff for surgical contraindication, this threshold aligns with previous reports indicating worse outcomes in cirrhotic patients with MELD ≥15 undergoing abdominal surgery. Therefore, a MELD score ≥15 may be considered as a marker of high-risk patients who require careful selection and optimization before hernia repair.**

**A limitation of our study is the relatively small sample size, which may have reduced the statistical power to detect certain differences, thereby increasing the risk of Type II error. Therefore, non-significant findings should be interpreted with caution, and future studies with larger cohorts are needed to validate our results. Given the retrospective design, small sample size, and potential selection bias, the conclusions drawn from this study must be interpreted with caution. Our findings are intended to highlight trends and generate hypotheses rather than establish definitive recommendations. Future prospective multicenter studies are needed to confirm these observations.**

**Although combining umbilical and inguinal hernia repairs in the analysis may introduce heterogeneity, we chose to include both due to the small number of eligible cirrhotic patients. Separate subgroup analyses were performed where feasible, but we acknowledge this as a limitation.**

In conclusion, our study demonstrates that mesh can be safely used in cirrhotic patients undergoing hernia repairs, without significantly increasing the risk of postoperative complications or mortality. However, the MELD score and emergency intervention remain an important predictor of survival, and patients with high MELD scores should be managed with caution. On the other hand, recurrence and ascites fistula are the most feared complications after hernia repair in these patients and mesh usage may increase the ascites fistula rate. Therefore, to find of true answer of this problem requires randomized prospective studies.

## Data Availability

The original contributions presented in the study are included in the article/Supplementary Material, further inquiries can be directed to the corresponding author.
